# Human norovirus persists longer than *Escherichia coli* in sandy soil, independent of plant decaying materials

**DOI:** 10.1038/s41598-025-31728-1

**Published:** 2025-12-15

**Authors:** Nuradeen Garba Yusuf, Courtney F. Aminirad, Kalmia E. Kniel, Sarah Strauss, Michelle D. Danyluk, Keith R. Schneider, Naim Montazeri

**Affiliations:** 1 Food Science and Human Nutrition Department, University of Florida - Institute of Food and Agricultural Sciences, Gainesville, FL USA; 2 Department of Microbiology and Cell Science, University of Florida - Institute of Food and Agricultural Sciences, Gainesville, FL USA; 3 Department of Animal and Food Sciences, College of Agriculture and Natural Resources, University of Delaware, Newark, DE USA; 4 Department of Soil, Water, and Ecosystem Sciences, Southwest Florida Research and Education Center, University of Florida - Institute of Food and Agricultural Sciences, Immokalee, FL USA; 5 Food Science and Human Nutrition Department, Citrus Research and Education Center, University of Florida - Institute of Food and Agricultural Sciences, Lake Alfred, FL USA; 6 Global Food Systems Institute - University of Florida - Institute of Food and Agricultural Sciences, Gainesville, FL USA

**Keywords:** Agricultural soil, Enteric pathogens, Food safety, Norovirus, Public health, Biotechnology, Microbiology

## Abstract

Human norovirus (HuNoV) is the leading cause of foodborne illnesses in the U.S. Fresh produce, often consumed raw, can serve as a vehicle for HuNoV transmission; however, limited data exist on its persistence in agricultural environments. This study evaluated the persistence of HuNoV GII, its cultivable surrogate Tulane virus, and *Escherichia coli* TVS 353 in sandy Florida agricultural soil. Soil samples with or without additional cilantro leaves (to simulate decaying plant debris) were incubated at 12 °C and tested for microbial concentrations at regular intervals over 29 weeks, using RNase RT-qPCR (both viruses), TCID_50_ (Tulane virus), and plate count (*E. coli*). Inactivation kinetics were fitted to log-linear and non-linear models to estimate the weeks required for the first 1-log_10_ reduction (*T*_*1*_*D*). Decaying cilantro leaves did not substantially impact microbial inactivation (*p* > 0.05). *E. coli* declined most rapidly (*T*_*1*_*D* = 2.2), followed by infectious Tulane virus (*T*_*1*_*D* = 5.63), Tulane virus genome copies (*T*_*1*_*D* = 11.8), and HuNoV GII genome copies (*T*_*1*_*D* = 28). A strong correlation of Tulane virus infectivity with HuNoV GII RNase RT-qPCR (*r* = 0.82) supported its suitability as a surrogate. Under the tested conditions, HuNoV’s prolonged persistence should be accounted for in risk assessments for preharvest fresh produce production.

**Supplementary Information:**

The online version contains supplementary material available at 10.1038/s41598-025-31728-1.

## Introduction

 Human norovirus (HuNoV) is the leading cause of enteric illness outbreaks globally^1^, accounting for the majority of foodborne illnesses in the U.S^2^. HuNoV is a non-enveloped, single-stranded positive-sense RNA virus from the *Caliciviridae*family^3^. Among the genetically diverse HuNoV strains, GII.4 has been consistently the most prevalent genotype associated with foodborne outbreaks in the U.S^4,5^. As an enteric pathogen, HuNoV transmits through the fecal-oral route, directly through person-to-person contact, and indirectly through consuming contaminated food or water. Its high infectivity, transmissibility, and environmental persistence make HuNoV a significant concern throughout the food supply chain^6,7^.

Fruits and leafy greens are high-risk foods linked to several HuNoV outbreaks^8,9^. These foods typically do not undergo a kill step prior to consumption; therefore, preharvest practices are crucial to preventing crop contamination during production. The use of organic matter, such as animal manure and sewage treatment residuals (i.e., municipal biosolids), to enhance the nutritional quality of soil for agricultural purposes can promote the growth and survival of pathogens, posing a significant risk of pathogenic contamination for produce^10–12^. Despite the public health burden of HuNoV as a leading foodborne pathogen, the risks associated with viral persistence in agricultural soil and subsequent transfer to produce remain understudied.

Previous studies have shown that norovirus can persist in soil for weeks to months, depending on extrinsic factors such as soil texture, organic matter, desiccation, and temperature^13–15^. Agricultural soil can serve as a vehicle for crop contamination with enteric pathogens when crops are grown directly in soil, during irrigation, rainfall events, or produce harvesting^12,16,17^. Therefore, assessing the prolonged persistence of HuNoV in agricultural soil is essential for informing produce safety guidelines and reducing the risk of foodborne transmission.

Enteric viruses, such as hepatitis A virus and HuNoV, have been isolated from naturally contaminated cilantro leaves^18^, indicating potential risks for pathogen transmission to humans and the potential for cross-contamination of other crops in the field. Whether decaying plant materials in soil contribute to HuNoV persistence remains unknown. This study evaluated the persistence of HuNoV in plant-decaying materials within agricultural soil. Because no culture-based method exists for absolute quantification of HuNoV, Tulane virus, a non-human calicivirus with genetic and structural similarities to HuNoV, was used as a cultivable surrogate alongside clinical HuNoV GII to assess infectivity^19^. Tulane virus is widely recognized as a suitable cultivable surrogate for HuNoV research for inactivation studies^19–21^. Viral quantifications were based on a molecular method (RT-qPCR) for both virus groups and a culture-based assay (50% tissue culture infective dose, TCID_50_) to estimate infectious Tulane virus particles^22^. The suitability of Tulane virus as a surrogate for HuNoV was evaluated by comparing inactivation kinetic parameters and the association between changes in their population densities over time.

*Escherichia coli* TVS 353 was included as a representative of enteric fecal bacteria, providing a practical reference for comparing bacterial and viral stability under the same conditions. Originally isolated from surface irrigation water, *E. coli* TVS 353 has been extensively used in studies on persistence and mitigation in agricultural water and soil^21,23–25^. Prior work comparing generic *E. coli* strains (TVS 353, 354, and 355) with pathogenic strains (O157 and non-O157) has shown that the generic strains exhibit greater persistence in soil environments, supporting their suitability as surrogates in field-based studies^26^. Overall, this study provides insights into the microbial hazard in produce-growing environments and supports risk-based strategies for improving prevention and mitigation of enteric pathogens in agricultural settings.

## Results

Weather conditions during soil collection and baseline soil characteristics are summarized in Table 1. No target microorganisms were detected in uninoculated samples. Headspace humidity within the sealed sample bags was not measured, as soil moisture is the primary factor influencing microbial stability and was measured in the study. Initial moisture content was slightly lower in soil-only samples (4.50 ± 0.52%) than in those with decaying plant materials (5.57 ± 0.32%), though the difference was not statistically significant (*p* = 0.083). Over 29 weeks, moisture content decreased by 0.66% in soil-only samples (*p* = 0.358) and 1.6% in soil with plant materials (*p* = 0.012), with no significant difference between matrices at week 29 (*p* = 0.534). Because the entire contents of each sample bag were analyzed, these minor moisture fluctuations were not expected to influence microbial counts per gram of soil.

**Table 1:** Weather conditions during soil sample collection and baseline soil characteristics.

Source	Parameter, measuring unit	Value
**Soil**	pH	6.95 ± 0.15
	Potassium (K), ppm	19.32 ± 0.10
	Ammonium (NH_4_N), ppm	0.32 ± 0.00
	Nitrate nitrogen (NO_3_N), ppm	1.51 ± 0.04
	Total Kjeldahl nitrogen (TKN), ppm	230.65 ± 7.53
	Total Phosphorus (P), ppm	325.03 ± 25.62
	Moisture content, %	4.50 ± 0.52
	Organic matter, %	1.37 ± 0.07
	Temperature, −10 cm depth (at the collection day)*, ºC	18.60 ± 2.17
	Temperature, −10 cm depth (7-day average)*, ºC	19.63 ± 1.82
**Weather**	Relative humidity, (7-day average)*, %	77.30 ± 17.35
	Solar radiation (7-day average)*,Watts/meter^2^	167.98 ± 249.86
	Temperature (at the collection day)*, ºC	16.00 ± 3.94
	Temperature (7-day average)*, ºC	18.34 ± 2.12

*Data were collected from the publicly accessible database UF/IFAS Florida Automated Weather Network (FAWN) from the Live Oak, FL station, adjacent to the sample collection site, URL: https://fawn.ifas.ufl.edu. All measurements are presented as mean ± standard deviation (*n* = 2), except for 7-day averages, which were calculated from hourly records collected daily.

Microbial recovery rates are shown in Table 2. Reported microbial concentrations do not account for potential losses during the recovery process. All microbial inactivation kinetic models converged, with goodness-of-fit provided in **Supplementary Table ****S1**. Using the best-fit models, the marginal means of the parameters were calculated and presented in Table 3. These models enabled comparisons across microbial groups and matrices, where applicable. Because the inactivation kinetics were comparable between the two matrices for all microbial targets, joint parameter estimates were also generated using the combined data.

**Table 2:** Percentage of microbial recovery rates across each assay and matrix (mean ± standard error, *n* = 3).

Microbe	Assay	Matrix	Recovery rate (%)
*E. coli* TVS 353	Plate count	Soil	41.65 ± 10.60
		Soil plus plant	38.63 ± 10.40
Tulane virus	TCID_50_	Soil	38.27 ± 9.57
		Soil plus plant	26.07 ± 6.52
HuNoV GII	RNase RT-qPCR	Soil	47.23 ± 15.05
		Soil plus plant	46.55 ± 12.08
Tulane virus		Soil	38.61 ± 10.66
		Soil plus plant	39.28 ± 12.59

**Table 3:** Best fitting models of microbial inactivation kinetics over time, with parameter estimates and the corresponding 95% confidence intervals [lower, upper].

Assay	Microbe	Matrix	Best fitting model	Parameters^1^	Matrix-specific estimates	Joint estimates
Plate count	*E. coli* TVS 353	Soil	*Weibull*	*T*_*1*_ *D*	2.13 [1.01, 3.25]	2.15 [1.32, 2.98]
				*β*	0.66 [0.53, 0.78]	0.65 [0.56, 0.74]
		Soil plus plant		*T*_*1*_ *D*	2.18 [0.96, 3.40]	
				*β*	0.64 [0.51, 0.77]	
TCID_50_	Tulane virus	Soil	*Weibull*	*T*_*1*_ *D*	5.52 [4.42, 6.84]	5.63 [4.84, 6.41]
				*β*	0.81 [0.72, 0.90]	0.82 [0.76, 0.88]
		Soil plus plant		*T*_*1*_ *D*	5.73 [4.62, 6.84]	
				*β*	0.83 [0.74, 0.92]	
RNase RT-qPCR	HuNoV GII	Soil	*Log-linear*	*T*_*1*_ *D*	26.4 [22.5, > 30.0]	28.1 [24.9, > 30]
		Soil plus plant		*T*_*1*_ *D*	29.7 [24.8, > 30.0]	
	Tulane virus	Soil		*T*_*1*_ *D*	11.3 [10.6, 12.0]	11.8 [11.3, 12.4]
		Soil plus plant		*T*_*1*_ *D*	12.4 [11.6, 13.3]	

^1^ *T*_*1*_*D*: the time in weeks needed to achieve the first 1-log_10_ reduction in the microbial population; and *β*, shape parameter. Extrapolations beyond 30 weeks were avoided to minimize uncertainty in model-based estimations. These values are provided in the full model output under the supplementary materials (10.5281/zenodo.16230597).

**Fig. 1:**
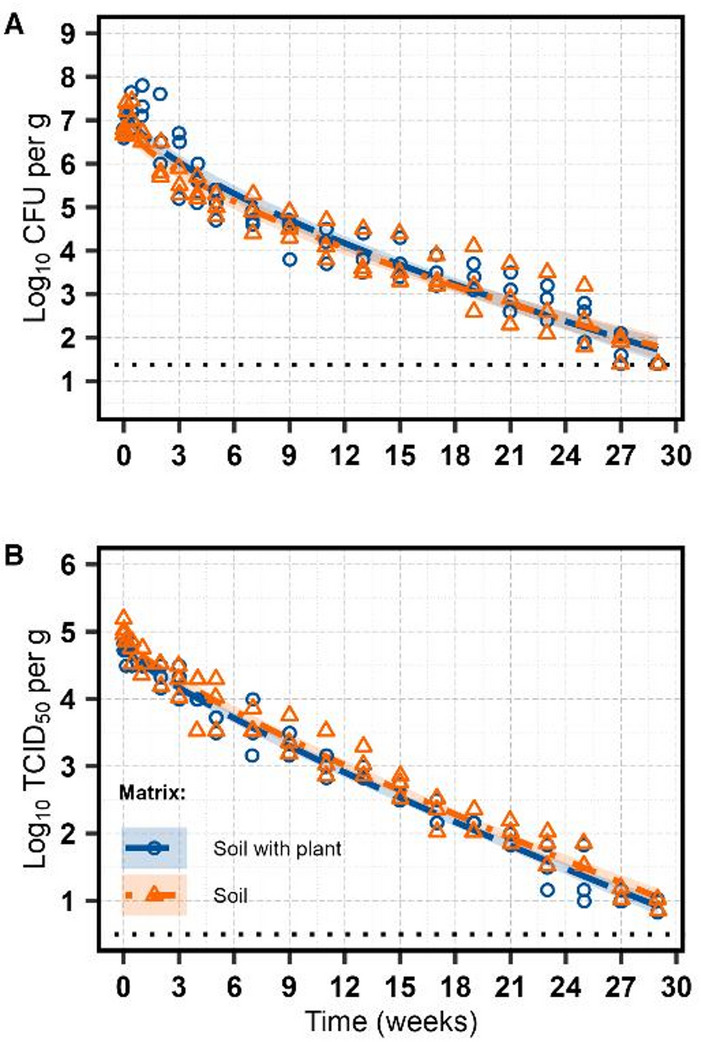
Inactivation of *E. coli* TVS 353 (**A**) and Tulane virus (**B**) in sandy soil plus plant and sandy soil-only sample over 29 weeks at 12 °C, quantified with total plate count and TCID_50_, respectively (*n* = 3). The best fit is illustrated by the dot-dashed and dashed lines for the soil-only sample and soil plus plant, respectively. Shaded areas represent 95% confidence intervals. The horizontal dotted line marks the detection limits of 1.38 log_10_ CFU/g for *E. coli* TVS 353 and 0.50 log_10_ TCID_50_/g for Tulane virus.

The inactivation kinetics of *E. coli* TVS 353 were best described by the Weibull model (Table 3; Fig. 1A). None of the bacterial counts reached below the detection limit of 1.38 log_10_ CFU/g over the 29-week study period. Estimated *T*_*1*_*D* values were similar in the soil plus plant materials (2.18 weeks [95% CI: 0.96, 3.40]) and soil-only samples (2.13 weeks [95% CI: 1.01, 3.25], *F*_*1, 113*_ = 0.004, *p* = 0.951), corresponded to a combined *T*_*1*_*D* value of 2.15 weeks [95% CI: 1.32, 2.98]. The shape parameter *β*, reflecting curvature of the Weibull model, did not differ significantly between matrices (*F*_*1, 113*_ = 0.035, *p* = 0.852) and was estimated at 0.65 [95% CI: 0.56, 0.74] for combined data. When models were refitted to estimate the time for the first two- and three-decimal reductions (*T*_*2*_*D* and *T*_*3*_*D*), the corresponding values across both matrices were 6.28 weeks [95% CI: 4.74, 7.81] and 11.70 weeks [95% CI: 9.79, 13.70], respectively.

Similar to *E. coli* TVS 353, Tulane virus infectivity, measured by TCID_50_ assay, was best fitted with the Weibull model (Table 3; Fig. 1B). No substantial heteroscedasticity was detected, so variance functions were not necessary. The estimated *T*_*1*_*D* value was 5.73 weeks [95% CI: 4.62, 6.84] in soil plus plant materials and 5.52 weeks [95% CI: 4.84, 6.84] in the soil-only samples, with no significant difference between matrices (*F*_1, 113_ = 0.07, *p* = 0.790), resulting in the combined *T*_*1*_*D* of 5.63 weeks [95% CI: 4.84, 6.41]. Furthermore, the respective *T*_*2*_*D* and *T*_*3*_*D* values were 13.1 weeks [95% CI: 12.1, 14.1] and 21.5 weeks [95% CI: 20.5, 22.5], indicating a slower viral decline than bacterial inactivation. The shape parameter *β* did not differ between matrices (*F*_1, 113_ = 0.08, *p* = 0.781), with a combined estimate of 0.82 [95% CI: 0.75, 0.88], indicating a moderately convex inactivation (tailing) curve. Although the concentrations stayed above the detection limit of 0.50 log_10_ TCID_50_/g, a tail-like persistence phase emerged near the end of the 29-week period.

**Fig. 2:**
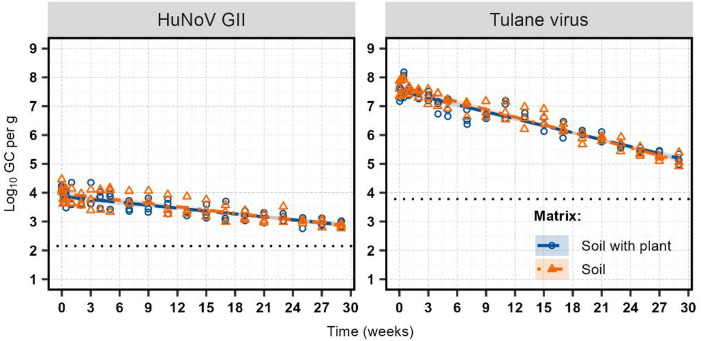
Inactivation of HuNoV GII and Tulane virus in sandy soil plus plant and sandy soil-only control samples over 29 weeks at 12 °C, quantified using RNase RT-qPCR (*n* = 3). Fitted values from the joint reparametrized log-linear models are represented as dashed lines for the soil plus plant sample and dot-dashed lines for the soil-only control sample. Shaded areas represent 95% confidence intervals. Dotted lines indicate the detection limits of 2.15 log_10_ GC/g for HuNoV GII and 3.78 log_10_ GC/g for Tulane virus.

Tulane virus and HuNoV GII were quantified using RNase RT-qPCR. Both remained detectable throughout the trials, and their inactivation kinetics were best described by the log-linear model by incorporating an exponential variance structure to improve the fit and correct the residual heteroscedasticity (Table 3; Fig. 2). Consistent with previous conditions, no significant difference in *T*_*1*_*D* values was observed across matrices (*F*_1, 230_ = 1.90, *p* = 0.173), corresponded to the estimated *T*_*1*_*D* values of 11.8 weeks [95% CI: 11.3, 12.4] for Tulane virus and 28.1 weeks [95% CI: 24.9, > 30] for HuNoV GII. The substantially higher *T*_*1*_*D* value for HuNoV GII reflects its greater persistence (*F*_1, 230_ = 98.50, *p* < 0.0001). The estimated *T*_*2*_*D* value was 23.7 weeks[95% CI: 22.58, 24.83] for Tulane virus, while HuNoV GII exceeded 30 weeks. For both viruses, achieving a *T*_*3*_*D* required more than 30 weeks.

We observed a nearly linear positive correlation between *T*_*1*_*D* and the *β* parameters in Weibull models for *E. coli* TVS 353 and Tulane virus infectivity. Parametric Monte Carlo simulations using the joint variance–covariance matrix (joint draws) confirmed that the observed correlation mainly affected the width of *T*_*1*_*D* confidence intervals, not central estimates. Independent-parameter simulations (assuming zero covariance) further inflated uncertainty, and in only one case, RNase RT-qPCR data for Tulane virus in the soil-only matrix, it produced negative bounds, though *T*_*1*_*D* estimates remained stable (**Supplementary Table S2**).

The log_10_ GC: TCID_50_ ratio was used to compare Tulane virus quantification by RNase RT-qPCR and infectivity assay. At inoculation, the ratio was 2.62 ± 0.10 in both matrices (*p* = 0.535), then increased over time in both matrices, reaching 4.18 ± 0.09 by the end of the study (*p* = 0.805). No main effect of matrix was observed (*F*_*1, 49.2*_ = 1.30, *p* = 0.263), but time had a significant effect (*F*_*19, 4.4*_ = 37.30, *p* = 0.001), indicating a progressive loss of infectivity relative to detectable virus particles with intact capsid. No significant matrix-by-time interaction was observed (*F*_*19, 4.4*_ = 1.10, *p* = 0.518).

**Fig. 3:**
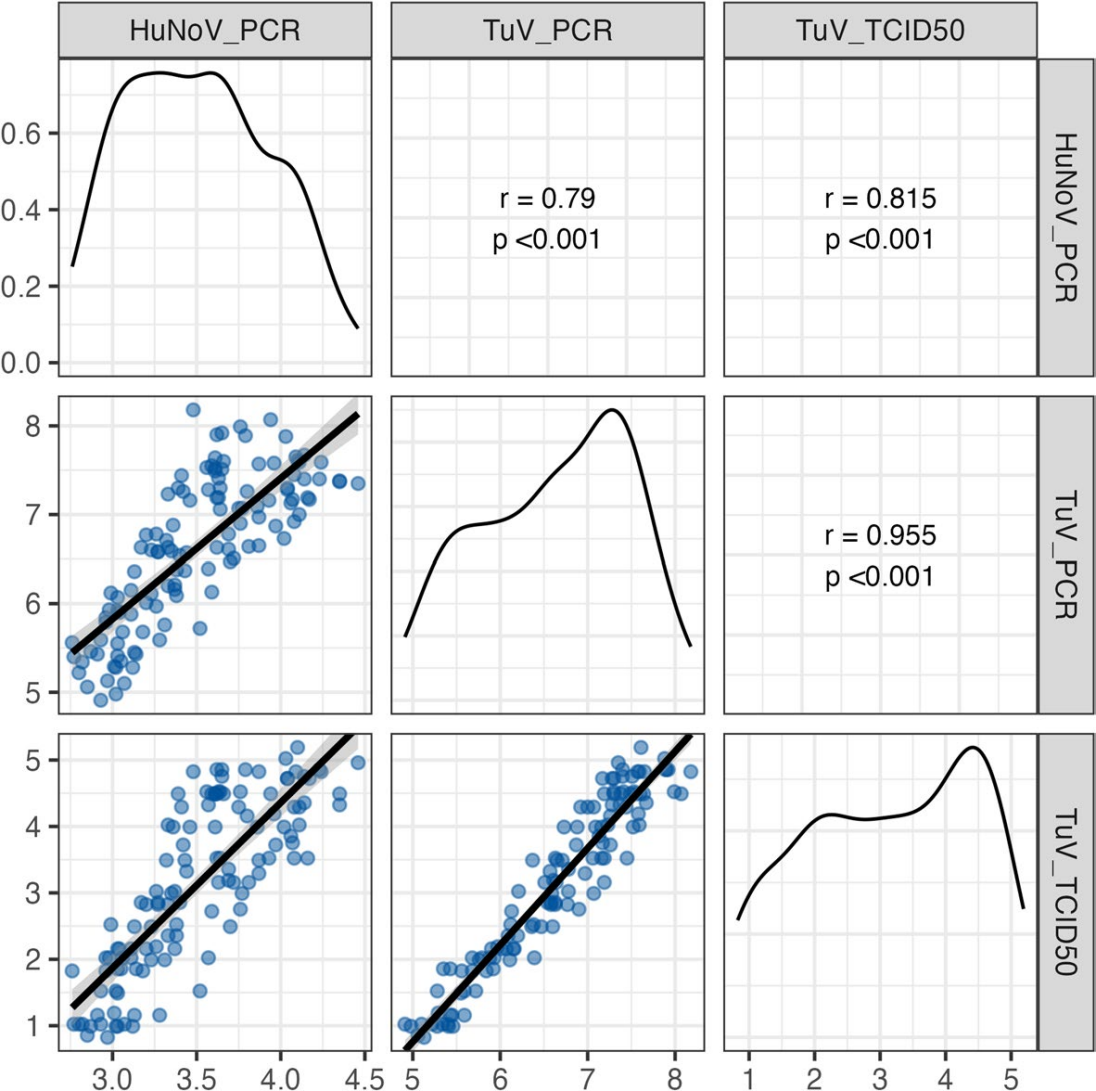
Pairwise correlation matrix of viral concentrations across experimental conditions (virus groups and assays). The lower triangle panels show scatterplots with fitted linear regression lines and shaded 95% confidence intervals. The upper triangle panels display Pearson’s product-moment correlation coefficients (*r*) with corresponding *p*-values. Diagonal panels present kernel density plots of viral concentrations for each condition.

To evaluate Tulane virus suitability as a surrogate for HuNoV GII, a Pearson correlation matrix was generated across virus types and quantification methods (Fig. 3). The strongest correlation was observed between the Tulane virus TCID_50_ assay and its RNase RT-qPCR measurements (*r* = 0.96, *p* < 0.001), indicating high consistency across detection platforms. A strong positive correlation was also found between the RNase RT-qPCR data for HuNoV GII and the infectivity assay for Tulane virus (*r* = 0.82, *p* < 0.001), suggesting similar behavior between the two viruses under the tested conditions. Tulane virus and HuNoV GII RNase RT-qPCR data showed a moderate correlation (*r* = 0.79, *p* < 0.001). Collectively, these findings support the use of Tulane virus infectivity data to track relative changes and trends in HuNoV.

## Discussion

The prolonged persistence of enteric pathogens in fecally contaminated soil increases the likelihood of crop contamination during preharvest stages^8,10–12^. To address the knowledge gap on persistence of enteric viruses in agricultural soil, we conducted a persistence study using HuNoV GII, its cultivable surrogate Tulane virus, and *E. coli* TVS 353, a non-pathogenic enteric bacterial model, in agricultural sandy soil with or without the addition of decaying plant materials. Microbial decay kinetics were modeled using linear and non-linear models. Deviations from log-linear inactivation are common in environmental matrices due to aggregation, adsorption, organic matter protection, or the presence of resistant subpopulations^27–29^. Non-linear models are increasingly favored as they better capture transition dynamics and provide more robust insights into pathogen survival than log-linear models, which often underestimate persistence at low concentrations^26,30^. We observed high correlations between Weibull parameter estimates, commonly reported due to mathematical dependencies between shape and scale parameters^31–33^. Although this dependency inflates parameter variances and covariances, the model can still produce nearly symmetric confidence intervals^31,32^. Incorporating the full variance–covariance matrix when computing estimated marginal means, as performed here, improves confidence interval accuracy, particularly under strong collinearity^32,34^.

The persistence of enteric pathogens in soil is shaped by both extrinsic and intrinsic factors, including soil texture, temperature, pH, salinity, organic content, microbial community composition, and the specific bacterial strain^27,35–37^. This study was conducted in highly sandy soil at a constant temperature of 12 °C in the dark. Storage below 8 °C or above 15 °C often results in two-stage (biphasic) kinetics, whereas intermediate temperatures tend to suppress this behavior^38^. A meta-regression analysis of studies on *E. coli* survival in soil at 4 °C to 38 °C showed that higher temperatures yielded a slower inactivation for nonpathogenic strains (0.90 log_10_ CFU per week) than for pathogenic strains (0.92 log_10_ CFU per week), indicating a moderate temperature effect^39^.

In this study, *E. coli* TVS 353 remained detectable throughout the 29-week period, and decaying plant material (cilantro leaves) had no significant effect on microbial persistence. Its inactivation in both matrices combined was best described by the Weibull model, with a combined *T*_*1*_*D* of approximately 2.2 weeks. This result agrees with reported *T*_*1*_*D* values of 2 weeks for *E. coli* on leafy greens^40^ and 2–8.6 weeks for *E. coli* O157:H7 in fresh produce growing soils^36^. A related laboratory-based study on*E. coli* O157:H7 strains in natural sandy soil from the same field reported a *T*_*1*_*D* of less than one week at 30 °C, with counts declining from approximately 7 log_10_ CFU/g to below the 0-log_10_ CFU/g limit of detection in 8 weeks^25^. In that work, autoclaving the soil reduced indigenous microbes and prolonged *E. coli* survival, with extinction (negative enrichment) occurring at week 24 versus 12 weeks in natural soil.

Although the bacterial population remained detectable throughout the study, the reduction observed in samples may partly explained by the ability of bacteria to enter a viable but non-culturable (VBNC) state. This adaptive mechanism, also documented for *E. coli* O157:H7, enables subpopulations to persist in a viable yet undetected form by standard culture-based methods^41^. Because no complementary viability assays were performed for *E. coli* TVS 353, the fraction of undetectable cells in a VBNC remains unknown. Consequently, the observed decline in culturable counts may not fully reflect the survival of viable cells, and the term persistence for *E. coli* in this context should be more precisely interpreted as culturability.

A 4-week study reported that *E. coli* TVS 353 in both amended and unamended soils from Beltsville, MD, U.S. (used during organic lettuce production in box planters at 20 °C) followed a log-linear model (*T*_*1*_*D* = 0.9 weeks), while *E. coli* O157:H7 strains were best described with Weibull^27^. That study also concluded biologically amended soil may support, but does not necessarily enhance, *E. coli* survival. Both strains showed *T*_*3*_*D* values within 3 weeks, whereas our data estimated 11.7 weeks to achieve a similar reduction. This finding is consistent with prior reports of greater persistence of TVS strains compared with pathogenic strains^26^.

Quantification of viral particles using culture-based and molecular methods, where applicable, provided insights into infectivity and the viral integrity in soil^19,42^. As shown with *E. coli* TVS 353, decaying cilantro leaves had no effect on HuNoV and Tulane virus persistence over 29 weeks. Faster reductions of murine norovirus (another HuNoV surrogate) and Tulane virus have been reported on spinach leaves grown in pots at 18 °C and 65% relative humidity (*T*_*1*_*D* of 0.7–0.8 weeks)^43^, and on the adaxial leaf surfaces of 4-week-old pre-harvest basil plants grown at 24 °C and 43% relative humidity (*T*_*1*_*D* of 0.4 weeks)^44^. These rapid declines were attributed to potential effects of plant-derived antiviral compounds, irreversible attachment of virus particles to plant-derived antiviral compounds, irreversible attachment, or internalization that prevented virus recovery for accurate enumeration^43,44^.

In contrast to *E. coli* TVS 353, both HuNoV and Tulane virus showed slower decay in soil samples. Tulane virus infectivity, measured by TCID_50_, declined with a *T*_*1*_*D* of 5.63 weeks, with *T*_*2*_*D* and *T*_*3*_*D* reached at 13.1 and 21.5 weeks, respectively. By comparison, murine norovirus in sandy and loamy soils showed faster inactivation by plaque assay, with *T*_*1*_*D* of approximately 2 weeks, followed by *T*_*2*_*D* of 3 weeks, and then reached the 1 log_10_ PFU/ml limit of detection in 5.5 weeks^15^. Another research on Tulane virus survival in gravelly loam soil demonstrated a 2–2.5 log_10_ reduction over 4 weeks, with a detectable virus titer by the end of the study period^45^.

This study used a TCID_50_ assay to assess changes in Tulane virus infectivity over time. Although widely applied, this method is generally considered less precise, offering lower sensitivity and no absolute quantification^22^. Nevertheless, Tulane virus remained detectable throughout the 29-week study, with levels above the 0.5 log_10_ TCID_50_/g detection limit. Using a log_10_ TCID_50_:PFU conversion ratio of − 0.65 [95%CI: −1.15, − 0.16]^22^, this limit corresponds to approximately 1.15 log_10_ PFU/g [95%CI: 0.66, 1.65]. Based on this conversion, the final titer of 0.96 log_10_ TCID_50_/g observed in both matrices equates to 1.61 log_10_ PFU/g [95%CI: 1.12, 2.11], approximating residual infectious particles near the tailing phase. Notably, the original ratio was derived from 96-well plate assays, while this study used 48-well plates, a methodological variation that may slightly affect conversion due to assay sensitivity.

RNase RT-qPCR quantification of Tulane virus yielded titers approximately 3.5 log_10_ units higher than TCID_50_, reflecting detection of both infectious and intact but non-infectious particles^22,46^. Persistence data obtained from RNase RT-qPCR were best fit by the log-linear decay model, which estimated longer *T*_*1*_*D* values of 28.1 weeks for HuNoV GII and 11.8 weeks for Tulane virus across both matrices. Because RNase depends on capsid degradation to expose viral RNA to enzymatic digestion, it cannot detect earlier mechanistic losses of infectivity, such as impaired receptor-binding^46^, likely explaining the slower observed inactivation kinetics. Virus aggregation or particle binding may also underestimate infectivity by altering apparent kinetics^29^, yet these particles can still be lysed during RNA extraction, allowing detection by RNase RT-qPCR. This independence from the particle state may partly explain the log-linear decay observed in molecular data.

Based on RNase RT-qPCR data, HuNoV GII required approximately 16.3 weeks longer than Tulane virus to reach the first log_10_ reduction, consistent with reviews reporting greater environmental resistance of HuNoV compared to its surrogates under similar stress^42^. Beyond intrinsic stability, HuNoV aggregation and particulate association matter in stool may contribute to its prolonged persistence in environmental matrices^29^. In contrast, monodispersed Tulane virus preparations used here^22^ may have enabled faster inactivation by enhancing virus exposure to extrinsic factors^29^, establishing a baseline for decay under the most vulnerable physical state. Other forms, such as vesicle-associated or cloaked clusters, may enhance stability; however, their protective role remains to be elucidated^47^.

This study observed a strong correlation between Tulane virus infectivity and RNase RT-qPCR data across both matrices (*r* = 0.96), indicating that RNase RT-qPCR reliably captured relative changes in Tulane virus persistence. A higher correlation (*r* = 0.99) was previously reported forserially diluted Tulane virus in buffer when comparing RNase RT-qPCR to plaque assay results^22^, likely due to the higher sensitivity of plaque assays and absence of virus-associated matrix effects. By contrast, the correlation between RNase RT-qPCR data for HuNoV GII and Tulane virus was moderate (*r* = 0.79), suggesting assay performance may vary by virus type. The correlation between HuNoV RNase RT-qPCR data and Tulane virus infectivity was slightly stronger (*r* = 0.82), reflecting a consistent association between the two assays in capturing overall inactivation trends.

Infectivity and RNase RT-qPCR data for Tulane virus suggest that prolonged persistence in soil, with or without plant materials, leads to loss of infectivity without major capsid degradation. These findings support the use of RNase pretreatment to improve the interpretation of RT-qPCR data for assessing virus viability. However, each method captures distinct aspects of virus integrity and must be interpreted within the context of its limitations^48^. As demonstrated here, strong correlations among quantification methods do not imply that the underlying inactivation kinetics, or the apparent decay rates, are directly comparable. To enhance biological relevance, future work should integrate viability assays and culture-based methods to strengthen the interpretation of persistence data and inform evidence-based guidelines for preventing viral contamination in fresh produce.

## Conclusions

This study addresses key gaps in the environmental stability of enteric pathogens relevant to preharvest produce safety. Using sandy soil from a Florida research farm, we evaluated the persistence of HuNoV GII, its surrogate Tulane virus, and *E. coli* TVS 353, while testing the influence of decaying plant material on inactivation kinetics. Molecular (RNase RT-qPCR) and culture-based (plate count and TCID_50_) quantification methods revealed distinct microbial- and assay-specific decay kinetics. Culture-based assays showed Tulane virus persisted much longer than *E. coli* TVS 353, highlighting the widely recognized environmental stability of enteric viruses. Under the tested conditions, Tulane virus served as a reliable surrogate for HuNoV GII, supported by a strong correlation between Tulane virus infectivity and HuNoV RNase RT-qPCR data. However, RNase RT-qPCR data suggested greater capsid stability for HuNoV GII relative to Tulane virus. Whether this structural integrity translates into prolonged HuNoV infectivity remains uncertain and warrants direct culture-based infectivity assays for HuNoV. Although conducted at a single temperature (12 °C) under controlled conditions (without sunlight or other environmental factors), this study provides valuable insights by using actual agricultural soil as the test matrix. Decaying cilantro leaves, at the tested levels, had no measurable effect on microbial decay. All microorganisms remained detectable throughout the 29-week period, signifying the potential for agricultural soils to serve as long-term reservoirs for enteric pathogens, particularly enteric viruses.

## Materials and methods

### Sample collection and soil quality analysis

Agricultural soil samples were collected in March 2024 from the University of Florida North Florida Research & Education Center – Suwannee Valley (Live Oak, FL, USA). The site was historically used for cultivating a variety of vegetables, including watermelon, tomatoes, and peppers. Weather and soil condition data were collected from the UF/IFAS Florida Automated Weather Network (FAWN) database, publicly accessible at https://fawn.ifas.ufl.edu.

All instruments were sanitized with 70% alcohol prior to use. Approximately 20 kg of soil was collected at a 15 cm depth and stored at 4 °C for up to one week before quality parameters were measured. To represent plant decaying materials, soil samples were tested for microbial persistence with and without the presence of cilantro (*Coriandrum sativum*) leaves. Fresh cilantro leaves with branches were purchased from local grocery stores in Gainesville, FL, and used without further washing.

Soil properties were measured on the original soil samples, except moisture content, which was determined in soil-only samples and those mixed with plant materials. Particle size distribution classified the soil as highly sandy^49^, with 94.9% sand, 3.1% silt, and 2.0% clay. Soil properties, except moisture content, were analyzed by the University of Florida IFAS Analytical Services Laboratory (Gainesville, FL). Soil pH was determined using the EPA Method 150.1 with an Orion Versa Star™ meter (Thermo Scientific, USA)^50^. Potassium was measured using EPA Method 200.7^51^ by inductively coupled plasma optical emission spectrometry (ICP-OES). Ammonium-nitrogen (NH_4_-N) was measured using a modified U.S. EPA Method 350.1^52^. Nitrate-nitrogen (NO_3_-N) was determined using U.S. EPA Method 353.2^53^. Total Kjeldahl nitrogen (TKN) was determined using U.S. EPA Method 351.2^54^ using an OI Analytical Flow Solution FS3700 Automated Chemistry Analyzer (Xylem Inc., Washington, DC). Total phosphorus was measured using U.S. EPA Method 365.1^55^, also with the OI Analytical Flow Solution FS3700 system. Soil moisture content was determined using a gravimetric method^56^, and organic matter content was assessed using the loss on ignition method described elsewhere^57^.

### Preparation of microbial stocks

Rifampicin-resistant *E. coli* TVS 353, originally isolated from surface irrigation water, was obtained from K. Schneider at the University of Florida (Gainesville, FL). Stock cultures were grown in brain heart infusion (BHI) broth (BD BBL™, Sparks, MD, USA) with 80 µg/mL rifampicin (TCI America™, St. Portland, OR, USA), and stored with 30% glycerol at − 80°C^25^. Working stocks were prepared from colonies grown on BHI-rifampicin agar, stored at 4 °C, and refreshed biweekly. For each inoculation, a single colony was transferred into BHI-rifampicin broth and incubated at 37 °C for 24 ± 2 h with shaking at 150 rpm. A 1 mL of culture was centrifuged at 8,000 ×g for 3 min, resuspended in 1 mL of phosphate-buffered dilution water (PBDW, pH 7.2; Alpha Biosciences, Baltimore, MD, USA), reaching a final concentration of approximately 9 log_10_ CFU/mL, and used for inoculation.

Tulane virus was provided by L.A. Jaykus at North Carolina State University (Raleigh, NC) and propagated in rhesus monkey kidney cells (LLC-MK2, ATCC CCL-7™) at a multiplicity of infection of 0.1. Infected cells were incubated at 37 °C with 5% CO_2_ in Opti-MEM™ medium (Gibco Life Technologies, Grand Island, NY, USA) supplemented with 2% fetal bovine serum (FBS, Gibco™, Thermo Fisher Scientific, USA), 1× antibiotic-antimycotic solution, 0.22% sodium bicarbonate, and 1× GlutaMAX™ (all from Gibco Life Technologies). Once cytopathic effects were observed, lysates were harvested, purified through iodixanol-based discontinuous gradient ultracentrifugation (OptiPrep™, Cosmo Bio USA, Carlsbad, CA), and resuspended in 10 mM Tris-1 mM EDTA buffer (pH 7.5), as described^22^. Purified stocks were stored at − 80 °C. Viral infectivity was later determined using the TCID_50_ assay as described below. The final suspension reached a titer of approximately 9.2 log_10_ TCID_50_/mL and was previously confirmed to contain primarily monodispersed particles^22^.

A diluted HuNoV GII-positive human stool specimen, from a symptomatic individual, was prepared as previously described^58^, served as the source of the virus stock. Presence of HuNoV GII at ~ 8.0 log_10_ Genome Copies (GC)/g was confirmed using the RNase RT-qPCR method described below. Briefly, the stool was suspended at a 1:5 ratio in PBDW (pH 7.2; Alpha Biosciences), vortexed at high speed for one minute, and centrifuged twice at 3,100 × g for 2 min. The clarified supernatant was aliquoted, stored at − 80 °C, and used as needed.

### Sample inoculation and storage

Experiments were performed in triplicate, with each microbial assay measured in duplicate. Ten grams of soil were placed in a 12.5 by 7.5 cm Whirl-Pak™ bag (Fisher Scientific, Waltham, MA, USA), which was equipped with a built-in leak-proof closure tab. A microbial cocktail was first prepared by combining 100 µL of Tulane virus working stock, 5µL of clarified HuNoV GII stool suspension, and 50 µL of *E. coli* TVS 353. Ten to thirteen cilantro leaves (approximately 0.75 g) were then each spot-inoculated with a 10-µL aliquot of the cocktail. Inoculating with a cocktail minimized potential matrix effects from the stool-derived HuNoV GII preparation and allowed a more consistent comparison of environmental stability across the three microorganisms.

Leaves were left under a biosafety cabinet for 60 min to allow the inoculum to dry and facilitate microbial attachment^59^, then were transferred to soil and hand-massaged for 30 s to settle the plant material at the bottom to decay. Inoculated soil with or without cilantro leaves contained initial concentrations of 5 log_10_ TCID_50_/g and 7.9 log_10_ GC/g for Tulane virus, 4 log_10_ GC/g for HuNoV, and 7 log_10_ CFU/g for *E. coli* TVS 353, as represented by week zero of the study period. For soil-only control, soil was inoculated with the same microbial cocktail, left for 60 min to permit adsorption, then mixed by hand. Microbial analyses were performed at days 0, 1, and 3, weekly from weeks 1 to 5, then biweekly from weeks 7 to 29. These time points allowed accurate kinetic modeling of microbial inactivation over time.

All bags were folded, sealed, and incubated under dark conditions at 12 °C in a refrigerated incubator for 29 weeks. This temperature reflects the lower bound of the U.S. FDA/EPA recommended water temperature range (12 °C and 32 °C) for testing sanitizer efficacy in preharvest agricultural water^60^ and matches conditions from a previous HuNoV and *E. coli* TVS 353 persistence study in preharvest water^61^. This temperature also approximates the average soil temperature of 18.2 ± 5.0 °C observed in Florida’s cool season from October to March (**Supplementary Fig. ****S1**)^62^, ensuring relevance to regulatory guidelines and supporting the integrated assessment of microbial risks in agricultural systems.

### Microbial recovery

Microbial recovery was performed based on the FDA Bacteriological Analytical Manual (BAM), which details the recovery of HuNoV and hepatitis A virus from green onions and leafy greens, after some modifications^63^. Major modifications included the change in elution buffer and the use of size exclusion filters instead of ultracentrifugation for the virus recovery. Briefly, the content of each sampling bag was transferred into a sterile 15 by 23 cm Whirl-Pak™ filter bag (Fisher Scientific), containing 30 mL of TGBE (pH 9.0) elution buffer, composed of 0.1 M Tris-base (Fisher Chemical, Ottawa, ON, Canada), 0.05 M glycine (Fisher Scientific, Geel, Belgium), and 10% (*w/v*) beef extract (Gibco Life Technologies, Detroit, MI, USA). Target organisms were eluted by mixing the content in a Stomacher^®^ 80 Biomaster (Seward Inc., NY, USA) for 30 s at the normal setting. To minimize debris carryover, the eluate was carefully recovered from the side of the filter bag and transferred into sterile tubes. A 1-mL aliquot of the eluate was immediately taken for *E. coli* TVS 353 enumerations by spreading 0.1 mL of undiluted and serially diluted samples on BHI-rifampicin agar, as explained earlier.

The remainder of the microbial elute was further processed for virus testing. First, samples were centrifuged at 12,000 × g for 20 min at 18 °C using an Allegra^®^ 64R benchtop centrifuge (Beckman Coulter Life Sciences, Indiana, USA). Then, the supernatants were concentrated using Amicon™ Ultra-15 Centrifugal Filter Units (MilliporeSigma™, Merck Millipore Ltd., Co Cork, Ireland) at 4,500 × g for 30 min at 18 °C before storage at − 80 °C. Prior to analysis, freshly thawed samples were sonicated in an ultrasonic bath at 150 W (40 kHz) for 4 min (Branson^®^, Model CPX1800H, Mexico) to reduce particle aggregation and virus-particle binding, as previously described^22,58^. This treatment did not adversely affect bacterial or viral viability. Microbial recovery rates (%) were determined at week zero by calculating the ratio of total microbes recovered to those added to samples.

### Microbial quantification

Samples were analyzed either undiluted or after serial dilutions. For the *E. coli* quantification, a 100 µL aliquot of each sample was plated onto BHI-rifampicin agar (Fisher Scientific) and incubated at 37 °C for 24 h. After incubation, visible colonies were enumerated. The detection limit was 1.38 log_10_ CFU/g. The infectivity of Tulane virus was assessed using the TCID_50_ assay, following a previously described method with minor modifications^22^. Virus samples were serially diluted in Opti-MEM™ medium (OptiPrep™, Cosmo Bio USA, Carlsbad, CA) supplemented with 1× antibiotic-antimycotic (Gibco Life Technologies, Grand Island, NY, USA), 2.94% (*v/v*) sodium bicarbonate (Gibco), and 1× GlutaMAX™ (Gibco Life Technologies). For each dilution, including undiluted samples, 120 µL was added to four replicate wells of a 48-well Nunc™ MicroWell™ plate (Thermo Fisher Scientific), each seeded with a 90% confluent monolayer of LLC-MK2 cells. Plates were incubated at 37 °C in a humidified 5% CO_2_ atmosphere for 1 h to allow virus adsorption, with gentle rocking every 15 min to ensure uniform distribution.

Following adsorption, 280 µL of Opti-MEM™ medium supplemented with 2% (*v/v*) FBS (Thermo Fisher Scientific) and an antifungal additive (Fungin™, InvivoGen, San Diego, CA, USA) was added to each well. Plates were incubated until cytopathic effects (CPE) were observed. Then, cells were fixed with 3.7% (*v/v*) formaldehyde (Thermo Fisher Scientific) and stained using 0.1% (*w/v*) crystal violet (Sigma-Aldrich, St. Louis, MO). Infectious virus titers, expressed as log_10_ TCID_50_/g, were calculated using the Reed-Muench method^64^, based on the proportion of wells showing CPE at each dilution. The assay’s detection limit was 0.50 log_10_ TCID_50_/g, corresponding to CPE observed in two of four replicate wells with undiluted samples.

To assess potential infectivity by targeting intact viral capsids, each 200-µL sample was treated with 1 U of RNase ONE™ (Promega, Madison, WI), as previously described^65^. Viral RNA was extracted using the QIAamp Viral RNA Mini Kit (Qiagen, Hilden, Germany), following the manufacturer’s instructions. Extracted RNA was analyzed in duplicate using the Luna^®^ Universal Probe One-Step RT-qPCR Kit (New England Biolabs, Ipswich, MA) on a CFX96 Touch™ Real-Time PCR System (Bio-Rad, Hercules, CA).

Each 25-µL RT-qPCR reaction contained 1× reaction mix, 1× enzyme mix, 25 U murine RNase inhibitor (New England Biolabs), 0.25 µM of each primer and probe, and 3 µL of RNA template. Separate assays were run for each virus using specific primers and probes: TVF/TVR/TVP for Tulane virus^66^ and JJV2F/COG2R/RING2P for HuNoV GII^67,68^. Thermal cycling conditions followed previously validated protocols^58^. Viral genome copies (GC) were quantified by generating standard curves from serial dilutions of RNA transcripts, in line with established methods^58^. To mitigate PCR inhibition, likely caused by co-extracted humic substances from soil and residual beef extract used during virus recovery^69^, all reactions were performed using a 1:10 dilution of the extracted template, and this dilution factor was incorporated into the final quantification. The detection limits were 3.78 log_10_ GC/g for Tulane virus and 2.15 log_10_ GC/g for HuNoV, based on a cycle threshold (Ct) cutoff of 40^22^.

### Statistical analysis and decay kinetics modeling

This study estimated microbial decay in soil plus plant (treatment) relative to soil-only (control). Estimated parameters are presented as marginal means ± standard error or with 95% confidence or quantile intervals [lower, upper], where applicable. Statistical analysis was conducted in RStudio version 4.5.0^70^. Data entry and initial preprocessing were performed using open-source R packages *tidyverse*^71^ and *here*^72^.

Inactivation kinetics of microorganisms were modeled jointly for each assay technique to allow statistical comparisons across microbial types and soil matrices, where applicable. Both linear and biologically relevant nonlinear models, the Weibull and log_10_-logistic, were fitted using generalized nonlinear least squares via the *nlme* package^73^. The log-linear model assumes first-order kinetics, assuming a constant microbial inactivation rate over time. Conversely, the Weibull model describes a continuous change in decay over time. The *β* parameter in a Weibull model distinguishes between linear (*β* = 1), concave (*β* > 1), indicating a tailing, and convex (*β* < 1), indicative of a shoulder^31^. The log_10_-logistic model assumes a log_10_-normal-like distribution of variability in microbial resistance to inactivation and has been used extensively in persistence and inactivation studies^21,74,75^. The reparametrized forms of the log-linear (Eq. 1) and Weibull (Eq. 2) models were based on previous studies^28,76^. The log_10_-logistic model (Eq. 3) was reparametrized according to our previous study^21^.

Unlike log-linear kinetics, decimal reduction time is not constant in non-linear models. Through reparameterization, we replaced the model’s decay rate with the *T*_*Δ*_*D* parameter, defined as the time in weeks required to achieve the first Δ-log_10_ reduction in microbial count. A larger *T*_*Δ*_*D* value, therefore, corresponds to slower decay and thus a longer persistence. The time for the first log_10_ (90%) reduction, *T*_*1*_*D*, was used as a comparative benchmark to evaluate initial decay across different microbial targets and matrices. The models were also refitted to estimate *T*_*2*_*D* and *T*_*3*_*D*, providing context on longer-term microbial decline. Because *T*_*Δ*_*D* alone cannot accurately estimate microbial population density at a given time, it is crucial to utilize the entire fitted model for prediction. Since the microbial inactivation kinetics may not remain consistent after 29 weeks, extrapolation beyond 30 weeks was avoided to minimize uncertainty in model-based estimations.

1 $$\:\:(\mathrm{l}\mathrm{o}\mathrm{g}-\mathrm{l}\mathrm{i}\mathrm{n}\mathrm{e}\mathrm{a}\mathrm{r}):{log}_{10}\:\left({N}_{t}\right)={log}_{10}\left({N}_{0}\right)-\:{\Delta\:}\:\left(\frac{time}{{T}_{{\Delta\:}}D}\right)$$

2 $$\:{\left(\mathrm{W}\mathrm{e}\mathrm{i}\mathrm{b}\mathrm{u}\mathrm{l}\mathrm{l}\right):\:log}_{10}\left({N}_{t}\right)={log}_{10}\left({N}_{0}\right)-{\varDelta\:\:\left(\frac{time}{{T}_{{\Delta\:}}D}\right)}^{\beta\:}$$

3 $$\:{(\mathrm{l}\mathrm{o}\mathrm{g}_{10}-\mathrm{l}\mathrm{o}\mathrm{g}\mathrm{i}\mathrm{s}\mathrm{t}\mathrm{i}\mathrm{c}):log}_{10}\left({N}_{t}\right)={log}_{10}\left({N}_{0}\right)-{log}_{10}\left(1+{e}^{\left(\frac{\mathrm{ln}\left(time\right)-\left[\mathrm{l}\mathrm{n}\right({T}_{{\Delta\:}}D)-\mathrm{l}\mathrm{n}({10}^{\varDelta\:}-1)\times\:{\upsigma\:}^2]}{{\upsigma\:}^2}\right)}\right)$$

where:

- *time*: incubation time (weeks),
- Δ: number of decimal reductions in microbial count,
- *T*_Δ_*D*: time in weeks required to achieve the first Δ log_10_ reductions in microbial count,
- *β*: shape parameter, and
- σ: variability in the population’s sensitivity to inactivation.

A null model was implemented by specifying constant parameters across the assays, assuming no effect of the matrix variable. This intercept-only model served as a baseline for comparison against more complex models incorporating matrix-specific parameters. The second-order Akaike’s Information Criterion (AICc) from the *performance* package^77^ was used to assess the goodness-of-fit, with penalties applied for models with more parameters to avoid overfitting. A model with an AICc at least two units lower than that of competing models was considered the best fit^78^. Model adequacy was further assessed by comparing residual diagnostic plots between the null and full models using the *ggResidpanel* package^79^, and variance functions from the *nlme* package were applied as needed to address heteroscedasticity^73^. The model with the lowest AICc and acceptable residual diagnostics was selected for statistical inference.

### Statistical inference and interpretation

Model coefficients and their associated uncertainties were estimated from the best-fitting models, followed by pairwise post-hoc comparisons and contrasts using the *emmeans* package^34^. Marginal means of estimated values across incubation weeks were determined with the *marginaleffects* package^80^. Because the fitted parameters showed notable correlation, a parametric Monte Carlo simulation was performed by drawing 5,000 coefficient vectors from the model-specific multivariate normal distribution using the *MASS* package^81^, preserving the full variance–covariance structure to assess its impact on level-specific *T*_*1*_*D* estimates. For each draw, *T*_*1*_*D* at each level (or level combination) was reconstructed from the appropriate linear combination of *T*_*1*_*D* coefficients; simulated means and 95% QIs were compared with the marginal means previously estimated from the established models. As a sensitivity analysis, the simulation was repeated with a diagonalized covariance matrix (off-diagonals set to zero) to approximate independent coefficients.

When applicable, observed data were subjected to pairwise comparisons across time points using the *multcomp* and *multcompView* packages^82,83^, with the significance level set at α = 0.05. Pearson’s correlation coefficients were calculated to assess similarities between virus groups and quantification methods. A correlation matrix was generated using the *GGally* package^84^. Additionally, the log_10_ GC: TCID_50_ ratio for Tulane virus was computed to assess the proportion of RNase RT-qPCR signalattributable to infectious particles^22^. This ratio supports the interpretation of molecular results in the context of risk assessment and validation of surrogate virus performance.

## Supplementary Information

Below is the link to the electronic supplementary material.

Supplementary Material 1

## Data Availability

The dataset generated and analyzed during the current study, along with the R scripts and their outputs, are available in the publicly accessible Zenodo repository at https://doi.org/10.5281/zenodo.16230597.
