# Supplementary material for: Human norovirus persists longer than Escherichia coli in sandy soil, independent of plant decaying materials
Source: Sci Rep. 2025 Dec 15;16:1935. doi: 10.1038/s41598-025-31728-1 (PMC12804744; doi:10.1038/s41598-025-31728-1)
Supplement: Supplementary file 1 — Supplementary Material 1 [file 41598_2025_31728_MOESM1_ESM.docx]

**Supplementary materials**

**
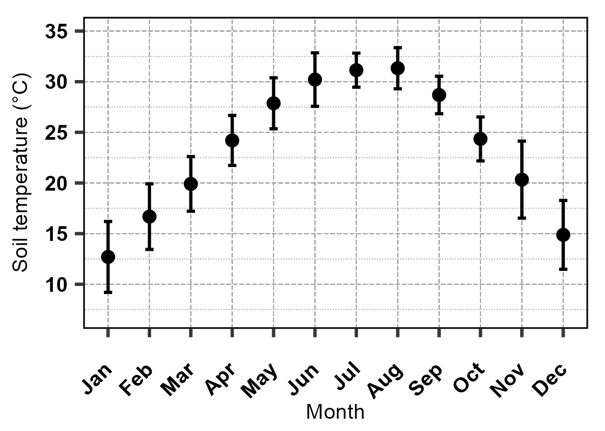
**

**Fig. S1***.* Monthly average soil temperature (10 cm depth) at the Live Oak, FL UF/IFAS Florida Automated Weather Network (FAWN) station adjacent to the sample collection site (mean ± standard deviation), calculated from daily averages for the period August 1, 2022–July 31, 2025. Data were obtained from the FAWN, URL: <https://fawn.ifas.ufl.edu/>.

**Table S1**: Model fit statistics (AICc and weights) for joint models fitted to observed data by assay across microbial groups and matrices, as applicable.

| Assay | Null model | Log-linear | Weibull | Log_10_-logistic |
| --- | --- | --- | --- | --- |
| Plate count | 201.0 (<0.001) | 202.7 (<0.001) | 168.9 (0.500) | 187.9 (<0.001) |
| TCID_50_ | -10.8 (<0.001) | -15.1 (<0.001) | -33.5 (0.500) | 52.2 (<0.001) |
| RNase RT-qPCR | 911.2 (<0.001) | 0.4 (0.474) | 6.2 (0.026) | 47.9 (<0.001) |

**Table S2:** Model-specific *T_1_D* estimates from parametric Monte Carlo simulation, based on joint (full) independent (diagonal) covariance matrices.

| **Assay** | **Microbe** | **Matrix** | **Estimates*** | |
| --- | --- | --- | --- | --- |
|  |  |  | **Joint** | **Diagonal** |
| *E. coli TVS 353* | Plate count | Soil plus plant | 2.12 [1.01, 3.23] | 2.14 [1.06, 3.22] |
|  |  | Soil | 2.18 [0.95, 3.37] | 2.19 [0.25, 4.14] |
| Tulane virus | TCID_50_ | Soil plus plant | 5.72 [4.62, 6.82] | 5.74 [4.68, 6.81] |
|  |  | Soil | 5.52 [4.41, 6.58] | 5.53 [3.67, 7.39] |
| HuNoV GII | RNase RT-qPCR | Soil plus plant | 29.70 [24.70, >30] | 29.70 [24.76, >30] |
|  |  | Soil | 26.40 [22.40, >30] | 26.40 [18.53, >30] |
| Tulane virus |  | Soil plus plant | 12.40 [11.60, 13.30] | 12.40 [5.31, 19.60] |
|  |  | Soil | 11.30 [10.60, 12.00] | 11.20 [<0, 22.80] |

* Estimates are means with 95% Monte Carlo simulation percentile intervals [lower, upper] from parametric multivariate-normal draws of the model coefficients (5,000 iterations). Negative *T_1_D* values occur only in the independent sensitivity and are nonphysical and are reported as *T_1_D* <0. Extrapolation beyond 30 weeks was avoided; therefore, they were reported as *T_1_D* >30. The underlying simulations were not truncated.
